# Structural Peculiarities of Ion-Conductive Organic-Inorganic Polymer Composites Based on Aliphatic Epoxy Resin and Salt of Lithium Perchlorate

**DOI:** 10.1186/s11671-017-2195-5

**Published:** 2017-06-20

**Authors:** Liubov Matkovska, Maksym Iurzhenko, Yevgen Mamunya, Igor Tkachenko, Valeriy Demchenko, Volodymyr Synyuk, Andriy Shadrin, Gisele Boiteux

**Affiliations:** 1 Institute of Macromolecular Chemistry of the NAS of Ukraine, Kharkivske shosse 48, Kyiv, 02160 Ukraine; 2 Université de Lyon, Université Lyon 1, Ingénierie des Matériaux Polymères, UMR CNRS 5223, 15 Boulevard A. Latarjet, Villeurbanne Cedex, 69622 France; 3 E.O. Paton Electric Welding Institute of the NAS of Ukraine, Kazymyra Malevych str. 11, Kyiv, 03680 Ukraine

**Keywords:** Epoxy resin, Lithium perchlorate salt, Glass transition temperature, Amorphous composite, Coordinative complexes, 81.07.Pr, 62.23.St, 66.30.hk

## Abstract

The article is concerned with hybrid amorphous polymers synthesized basing on epoxy oligomer of diglycide aliphatic ester of polyethylene glycol that was cured by polyethylene polyamine and lithium perchlorate salt. Structural peculiarities of organic-inorganic polymer composites were studied by differential scanning calorimetry, wide-angle X-ray spectra, infrared spectroscopic, scanning electron microscopy, elemental analysis, and transmission and reflective optical microscopy. On the one hand, the results showed that the introduction of LiClO_4_ salt into epoxy polymer leads to formation of the coordinative metal-polymer complexes of donor-acceptor type between central Li^+^ ion and ligand. On the other hand, the appearance of amorphous microinclusions, probably of inorganic nature, was also found.

## Background

Liquid electrolytes are commonly used in lithium or lithium-ion batteries at room temperature with the ionic conductivity from 10^−3^ to 10^−2^ S/cm [1, 2].

It would be useful if the batteries could work at higher temperatures, since it will be no need to use a separate cooling circuit at the system level, or such need will be significantly reduced [3]. However, high temperatures create difficulties because of quick degradation of liquid electrolytes [3]. In addition, there are two main problems that hinder the development of liquid electrolytes. First, formation of the lithium dendrites on electrodes leads to serious danger due to the potential possibility of internal short circuits. Second, the electrochemical instability of the lithium electrodes causes an insignificant life cycle of batteries during repeated processes of charging/discharging [4].

In addition, the first generation of battery prototypes, which used liquid electrolytes, have a high risk of leaks affecting on the reliability of a device [1]. Safety is one of the most pressing issues related to the further progress in development of the next-generation batteries. That makes solid electrolytes one of the most promising candidates for replacement of flammable and potentially dangerous liquid electrolytes [5].

Solid polymer electrolytes (SPE) have been used in different applications as the ion conductors in various electrochemical devices such as lithium batteries, ultracapacitors, fuel cells, and solar cells [6]. Rechargeable lithium and lithium-ion batteries play an important role on the market of electrochemical energy storage devices, since they are widely used for charging of portable electronic devices and for autonomous controlling devices [2]. Therefore, recently, the development of new solid polymer electrolytes was an important objective, since optimal balance between high ionic conductivity and technological conditions of material has not been still achieved [7, 8]. SPE have such properties as good compatibility with electrodes, low self-discharging rate, easy processing for various shapes and sizes, lack of leakage, flexibility, and self-sufficiency for form changes during charge-discharge cycles [7–10].

Polyethylene oxide (PEO) [11, 12] is one of the mostly studied oligomers, which are used for SPE creation due to the effective coordination of metal ions in it because of the optimum distance and orientation of ether oxygen atoms in its molecular chains [12]. The disadvantage of PEO is amorphous-crystalline structure [4, 13, 14] that leads to the conductivity through the amorphous area of a polymer only [11, 15] above the glass transition temperature *T*_g_ [6, 12, 16], and as a result, PEO has low ionic conductivity at room temperature because of the presence of high crystalline phase [4, 14, 17].

Nowadays, as a rule, solid polymer electrolytes include inorganic salts dissolving in oligomers which, in their turn, form a solid matrix with the ionic conductivity [10, 12, 18, 19]. Added salt serves as a source of ions and contributes their movement along the polymer chains, so that it plays the crucial role in ion transport in polymer electrolytes [16]. Hereby, concentration and mobility of ions are significant parameters affecting the conductivity in polymer electrolytes [14, 17]. Understanding of mechanism of the ion transport in a polymer requires the study of ion-ion and ion-polymer interactions that is of great interest [10, 14]. Many studies of ion transport in polymer electrolytes have been conducted using various types of cations such as Na^+^, Li^+^, Ag^+^, and Mg^+^ [20]. However, the composites based on lithium salts are preferably studied, because the Li^+^ cations are the smallest and can easily move in a polymer matrix [17, 20]. Another important characteristic is thermal stability of ions and their inertness to the cell components [21].

High ionic conductivity can be achieved by increasing the salt concentration in polymer [6]; however, the authors [9] have shown that conductivity of the composites based on PEO is limited by certain value of a salt concentration. At higher salt concentration, the conductivity decreases because of formation of the ion complexes, which, in turn, causes reduction of the ionic mobility and the number of charge carriers [10].

For application as electrolytes, polymers should have certain properties, such as amorphousness, the presence of ether oxygen in their structure, low glass transition temperature, high-dimensional stability, mechanical strength, and the ability to form thin films [9, 18]. One of the suitable materials that satisfies these requirements is aliphatic epoxy oligomer, namely, the diglycide aliphatic ester of polyethylene glycol. It has an identical to polyethylene oxide chain structure, however, is amorphous and is able to dissolve the high concentration of lithium perchlorate salt similarly to PEO.

Therefore, the aim of the present work is the synthesis of solid amorphous polymer composites based on aliphatic epoxy oligomer and the study of influence of lithium perchlorate salts on their structure.

## Methods

### Materials and Synthesis

The epoxy oligomer (diglycide aliphatic ester of polyethylene glycol (DEG-1)) and lithium perchlorate (LiClO_4_) salt were used for synthesis of ion-conductive epoxy polymer composites. These components were previously pre-dried in vacuum at 80 °C during 24 h. After drying, the salt was dissolved in the DEG-1 oligomer. Solutions of DEG-1-LiClO_4_ were prepared with LiClO_4_ content from 0 to 50 phr of DEG-1. 10 phr of polyethylene polyamine (PEPA) hardener has been used as a curing agent for synthesis of the composites.

Thermal characteristics were studied by differential scanning calorimetry (DSC) with TA Instruments DSC Q2000 in the temperature range from −70 to +150 °C with the heating rate of 10 °C/min. Glass transition temperature (*T*_g_) was determined from the DSC curves at the second heating. The experimental error of determination of the glass transition temperatures was ±1 °C.

The electrical and dielectric characteristics were investigated by the broadband dielectric analyzer “Novocontrol Alpha” with Novocontrol Quatro Cryosystem (Novocontrol Technologies, Montabaur, Germany) that was equipped with a two-electrode circuit, in the frequency range from 10^−1^ to 10^7^ Hz and the temperature range from −60 to +200 °C. The voltage applied to a sample was equal to 0.5 V. The test samples had a diameter of 20 mm and a thickness of 0.5 mm and were previously coated by aluminum layer under vacuum. The obtained data was analyzed using the software “Novocontrol WinDeta 3.8.”

Structural organization and features of macromolecular ordering of the synthesized polymer systems were investigated by wide-angle X-ray spectra (WAXS) using the X-ray diffractometer DRON-4.7. X-ray optical scheme was performed by Debye-Scherer method on passing the primary beam through the polymer sample polymer using CuK_*α*_ emission (*λ* = 1.54 Å) that was made monochromatic by Ni filter. The investigations were carried out by automatic step scanning in the range of scattering angles (2*θ*) from 2.6° to 40°, and the exposure time was 5 s.

Infrared (IR) spectroscopic studies have been performed using spectrometer with Fourier transformation “Tensor-37” from Bruker Corp. in the range of wave numbers 600°–3800° cm^−1^. According to the passport of the device, the relative measurement error is <2%.

The morphological features of the synthesized composites were studied using method of the reflective optical microscopy (ROM) by Unicorn NJF 120A polarization microscope at polarization angles 0°–90°. Microphotographs analysis was performed using Carl Zeiss Imaging Solutions AxioVision V4.7.1 software.

Structural features of the synthesized composites have been studied using methods of the electron microscopy (SEM) by JEOL 100-CX II transmission electron microscope, equipped with scanning system. Elemental analysis of inclusions was performed using analytical complex consisting of a scanning electron microscope JEOL JSM-35CF, and X-ray spectrometer with dispersive in energy X-ray quanta (Model INCA Energy-350 from “Oxford Instruments”). An important feature of such electron microprobe analysis is its locality: maximum size of the excitation area is 2 μm. According to the morphological characteristics and the chemical composition basing on the energy dispersive spectral analysis, the automatic separation of inclusions on types (elemental composition) and measuring of their sizes has been carried out. The results have been analyzed using a special program for quantitative phase distribution and inclusion investigation. All results are presented in weight percent. The experimental error was 0.1%.

WAXS, IR, ROM, and SEM studies and elemental analysis were carried out at the temperature *T* = 20 ± 2 °C.

## Results and Discussion

**Fig. 1:**
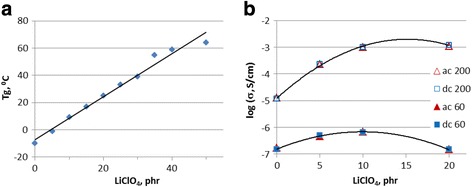
Thermal and electrical characteristics of composites. **a** Dependence of glass transition temperature *T*_g_ (**a**) and conductivity *σ* at 60 and 200 °C (**b**) on LiClO_4_ salt content

Analysis of the DSC studies as well as of the dielectric and electrical investigations of the synthesized composites with lithium perchlorate salt content from 0 to 20 phr was previously presented in our paper [22]. Further widening of LiClO_4_ content (from 0 to 50 phr) maintains linear increase of glass transition temperature *T*_g_ from −10 to 64 °C (Fig. 1a). That can be a result of electrostatic interactions between lithium cations Li^+^ and the macromolecular chain of DEG-1 with forming of coordinative complexes, which are accompanied by displacement of electron density of the oxygen atoms and their partial polarization. It is reflected in a substantial reduction of segmental mobility of DEG-1 chains within the formed complexes that is shown up in the increase of glass transition temperature of polymer matrix.

Figure 1b shows the change of conductivity *σ* with growth of LiClO_4_ content in composite. At low temperature (60 °C), the maximal value of *σ* is reached at 10 phr of salt and the *σ* value of composite with 20 phr of LiClO_4_ is equal to that in pure DEG-1. At high temperature (200 °C), the *σ* values are three orders of magnitude higher with maximum at 15 phr of LiClO_4_. Such character of conductivity dependence on LiClO_4_ content can be explained by existence of two opposite competitive processes. First, growth of salt content in composite gives the increase of carrier number and the raise of conductivity. Second, growth of *T*_g_ reflects the restriction of DEG-1 molecular movements which reduces the carriers mobility. At higher temperatures, the raising of molecular movements compensates this mechanism and conductivity becomes essentially higher.

**Fig. 2:**
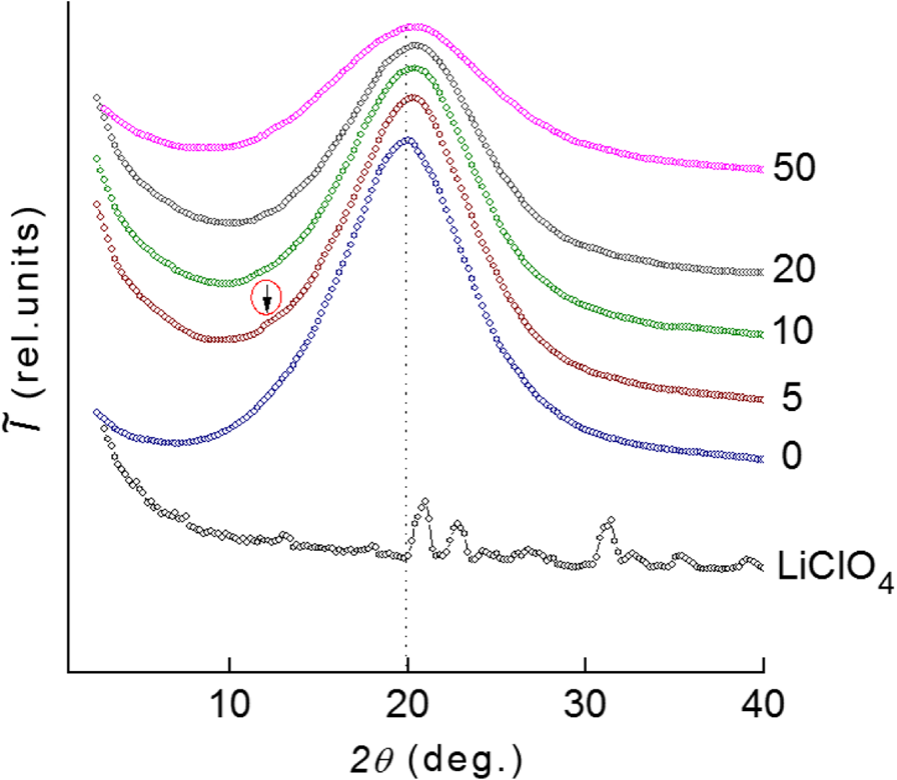
The WAXS studies of the DEG-1/LiClO_4_ systems. The wide-angle X-ray diffraction patterns of a lithium perchlorate salt LiClO_4_ and systems with the different salt content (shown near curves)

Analysis of the wide-angle X-ray diffraction patterns of the systems has shown that all of them are amorphous (Fig. 2). The average value of the period (*d*) of a short-range molecular ordering of DEG-1/PEPA internodal molecular segments located in the polymer volume can be calculated using Bragg equation:

$$ d= l{\left(2 \sin {\theta}_m\right)}^{-1} $$

where *λ* is the wavelength of the characteristic X-ray emission (*λ* = 1.54 Å for CuK_*α*_ emission) and it equals to 4.44 Å.

However, the introduction of LiClO_4_ salt having a crystalline structure into the epoxy resin is accompanied by changes in the diffraction pattern. That is evidenced by the presence of subtle diffraction peak of the diffuse type at 2*θ*_*m*_ ≈ 12.2° on the background of the amorphous halo, which is similar to the angular position of the DEG-1 at 2*θ*_*m*_ ≈ 20.0° (*d* ≈ 4.39 Å). This diffraction peak characterizes the existence of metal-polymer complexes of the donor-acceptor type, in our case, between central ions (Li^+^) and ether oxygen of the epoxy chains in the intermolecular volume of the epoxy resin, and this confirms the assumptions made by the analysis of DSC data. Basing on the angular position of this diffraction peak in composite with 50 phr of LiClO_4_, the average Bragg distance *d* between the molecular chains coordinated by cations Li^+^ is 4.30 Å.

Structure of the polymer composites has been investigated by means of infrared spectroscopy. The main absorption bands of LiClO_4_, DEG-1, and PEPA with relevant groups are presented in Table 1. These absorption bands were interpreted in accordance with [23–25], respectively.

**Table 1:** Interpretation of the absorption band IR spectra of LiClO_4_, DEG-1, and PEPA

LiClO_4_		DEG-1		PEPA	
Wave number, cm^−1^	Group, type fluctuations	Wave number, cm^−1^	Group, type fluctuations	Wave number, cm^−1^	Group, type fluctuations
3100–3600	–OH, *ν* (a water)	3100–3600	–OH, *ν*	3100–3600	NH_2_ + NH, *ν*
1637	LiClO_4_	2914	–CH_2_–, *ν*_asym_	2941	–CH_2_–, *ν*_asym_
1086, 1113, 1146	ClO_4_^−^, *ν*_asym_	2872	–CH_2_–, *ν*_sym_	2827	–CH_2_–, *ν*_sym_
941	ClO_4_^−^, *ν*_sym_	1458	–CH_2_–, *δ*	1641, 1585	NH_2_ + NH, *δ*
627	ClO_4_^−^ (not associated with Li^+^)	1352	CH_2_–, *δ*	1462	CH_2_–, *δ*
–	–	1253	Epoxy group, *ν*_sym_	1310	CH_2_–, *δ*
–	–	1105	C–O–C, *ν*	1124	C–N–C, *ν*
–	–	910, 856	Epoxy group, *ν*_asym_	–	–

*ν* valence vibration, *ν*_sym_ and *ν*_asym_ valence symmetric and asymmetric vibrations, *δ* bending vibration

**Fig. 3:**
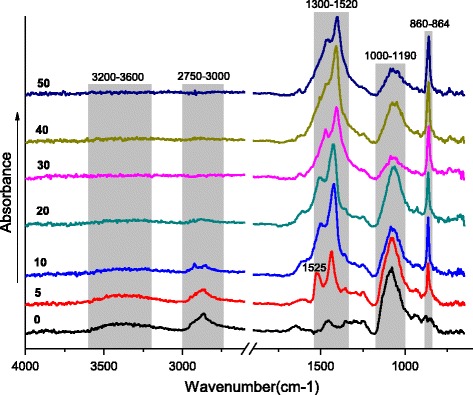
The IR spectra of the composites. The infrared spectroscopy of the systems with the different lithium perchlorate salt content (the numbers near curves)

**Fig. 4:**
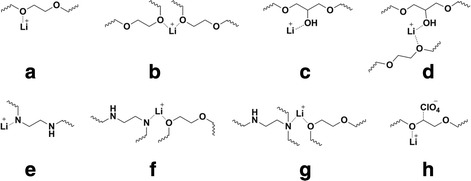
**a**–**h** Schemes of complexes. Complexes which possibly exist in the systems LiClO_4_/DEG-1/PEPA

As one can see, the characteristic absorption bands of epoxies’ ring are absent in the spectra (Fig. 3, 0 phr LiClO_4_ content) that indicates the complete curing of epoxy component. These absorption bands are also absent in the IR spectra (Fig. 3, 5–50 phr) of the cured composites. The absorption bands in the range of wave numbers 1300–1520 and 1000–1190 cm^−1^, which respectively correspond to fluctuations of –CH_2_– and (C–O–C and C–NC) groups, expand and shift to the low-frequency region with LiClO_4_ content increase. It is known that this can be associated with the formation of coordination bonds between Li^+^ cations and ClO_4_^−^ anions and polymer chains [26, 27]. It is generally accepted that Li^+^ cations can easily form complexes with polyethylene ether bonds [23, 24, 27–30] as well as with polyamines [31]. The absorption band at 1637 cm^−1^ in the IR spectrum of LiClO_4_ indicates its undissociated state (Table 1) [23, 24]. It should be noted that this band in the IR spectra of 5–50 samples is absent. This indicates that the pure (undissociated) form of LiClO_4_ in the composites is not contained. In accordance to this, in Fig. 4 the possible ion-dipole interactions of Li^+^ ion with the ether bond of polyethylene oxide fragment and OH group of the disclosed epoxy ring of DEG-1 (Fig. 4a–d) and with secondary amine group of PEPA (Fig. 4e), with secondary or tertiary amine group and ether bond simultaneously (Fig. 4e–g), are shown. As an example of coordinated ClO_4_^−^ ion, Fig. 4h represents the scheme of this anion interaction with positively charged carbon atom that is located near the electronegative oxygen atom.

**Fig. 5:**
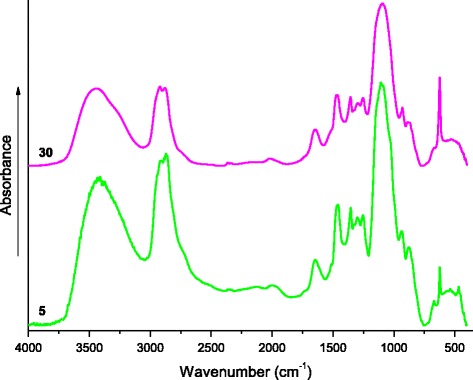
The IR spectra (in tablets of KBr). The IR spectra of the composites with different LiClO_4_ content (marked by numbers near curves)

It should be noted that in the sample with addition of 5 phr lithium perchlorate and after solidification, a new absorption band at 864 cm^−1^ appears in its IR spectrum, which maintains in the samples with 10–50 phr salt content. Considering that the most of the absorption bands of metal complexes are located in the low-frequency region [32], it is obvious that this absorption band is associated to complexes involving LiClO_4_. The interaction of PEO and LiClO_4_ is widely studied in literature, and in presented materials, the absorption band around 860 cm^−1^ is absent [23, 24, 27–30]. Probably, this band refers to formation of lithium amino-complex, which presence influences the fluctuations of methylene groups located nearby. That is confirmed by appearance of a new absorption band in the IR spectrum of composite with 5 phr of LiClO_4_ at 1525 cm^−1^ (Fig. 3), which is shifted to the low-frequency region with increasing of the salt content up to 50 phr. In accordance to [26], this is due to the increasing number of coordination bonds. Important to note that when film samples with 5–50 phr of LiClO_4_ were crushed into a powder and molded in the KBr tablets, the described absorption bands at ≤1525 and 860–864 cm^−1^ disappeared, since crushing leads to destruction of the weak coordination bonds. That also confirms the coordination nature of these bands. As an example, Fig. 5 shows the IR spectra of the samples with 5 and 30 phr of LiClO_4_ contents.

Generally, the increasing quantity of LiClO_4_ leads to the increase of the coordination bonds, which reduce mobility of macromolecular chains [28]. Figure 3 shows a gradual decrease in intensity of the absorption bands associated with valence fluctuations of OH–, NH–, and –CH_2_– groups. At the destruction of coordination bonds in the samples molded in the KBr tablets, these valence bonds are clearly manifested (Fig. 5).

In addition to the coordination bonds, other important factors which influence on the structure of the obtained composites should be indicated. Thus, the reaction between DEG-1 and PEPA in the presence of LiClO_4_ occurs more completely, apparently (to a certain limit of salt), since it is known that LiClO_4_ is an effective aminolysis catalyst of oxiranes ring [33]. Another structure-forming factor can be realized as a net of hydrogen bonds, including ones with the participation of ClO_4_^−^ ions [30].

In our case, the complexes of Li^+^ ions with maximum coordination number 2 are presented in Fig. 4, but the coordination number of ions can reach 8 [31]. Due to small radius (0.6 A), the Li^+^ ions are highly mobile, so they can create as well as destroy the complexes easily [29, 31], and because of that, it is difficult to define complex structure involving lithium perchlorate salt that was formed during the curing reaction.

**Fig. 6:**
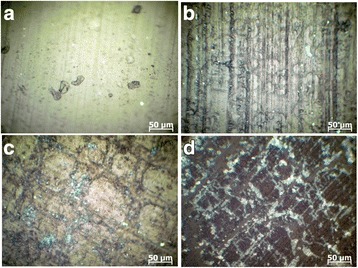
Reflective optical microscopy results. ROM of the epoxy polymer with **a** 0, **b** 10, **c** 20, and **d** 50 phr LiClO_4_ salt

**Fig. 7:**
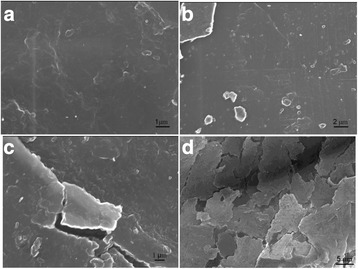
Scanning electron microscopy results. SEM of the composites with **a** 0, **b** 10, **c** 20, and **d** 50 phr LiClO_4_ salt

**Fig. 8:**
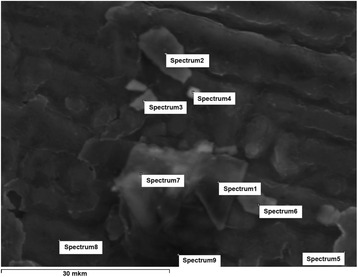
SEM with elemental analysis. SEM of the composite with 50 phr LiClO_4_ surface with the appointment of elemental analysis spectra

Figure 6 presents the micrographs of reflective optical microscopes of the synthesized composites with different content of lithium perchlorate salt. The formation of ordered structures in the composites with addition of the salt into the system was observed. In this case, the reflective optical microscopy used polarized mode revealed the presence of the distributed inclusions, probably, of inorganic nature with sizes ranging from 2 to 20 μm. In order to confirm the presence of inclusions in the composites, the structural investigation that used scanning electron microscopy was conducted. The results are presented in Fig. 7. One can see the presence of the inclusions observed by ROM (for the samples with some LiClO_4_ content) and the increase of their number and size with the increase of LiClO_4_ content in the composites. In order to determine the nature of found inclusions, the elemental analysis of nine different areas of surface of the composite with 50 phr LiClO_4_ has been fulfilled (Fig. 8). Normalized mass distribution of elements in the microareas shown in Fig. 8 are presented in Table 2. It is evident that content of elements in spectra is different. It is important that the inclusions identified by ROM (Fig. 6) and SEM (Fig. 7) are characterized with the decreased content of carbon and the increased content of oxygen and chlorine, which enter into the composition of LiClO_4_ (spectra 3–5), comparing to the spectra of the polymer matrix (spectra 7–9). That can be explained by the oxygen and chlorine atom aggregation and, perhaps, also with aggregation of lithium atoms (however, it was impossible to determine such aggregation with the conducted investigations) from the lithium perchlorate salts dissolved in DEG-1 during its synthesis. The presence of a number of carbon atoms (even in the spectrum 3–5 of the inclusions) can be explained by the overlapping of their high content in macromolecular chains of polymer matrix that may partially cover the inclusions.

**Table 2:** The mass distribution (%_wt_) of elements in different areas of the composite surface with 50 phr of LiClO_4_

Spectrum	C	O	Cl	Total
Spectrum 1	68.8	25.2	6.0	100.0
Spectrum 2	34.9	62.9	2.2	100.0
Spectrum 3	52.8	36.9	10.3	100.0
Spectrum 4	61.6	29.8	8.6	100.0
Spectrum 5	55.2	39.8	5.0	100.0
Spectrum 6	40.9	56.2	2.9	100.0
Spectrum 7	55.1	42.9	2.0	100.0
Spectrum 8	54.7	41.2	4.1	100.0
Spectrum 9	58.8	37.3	3.9	100.0

The elemental composition of the initial lithium perchlorate salt was also determined. It was found that the chlorine in salt is 41.61%_wt_ and oxygen is 58.39%_wt_. Lithium content could not be determined.

**Fig. 9:**
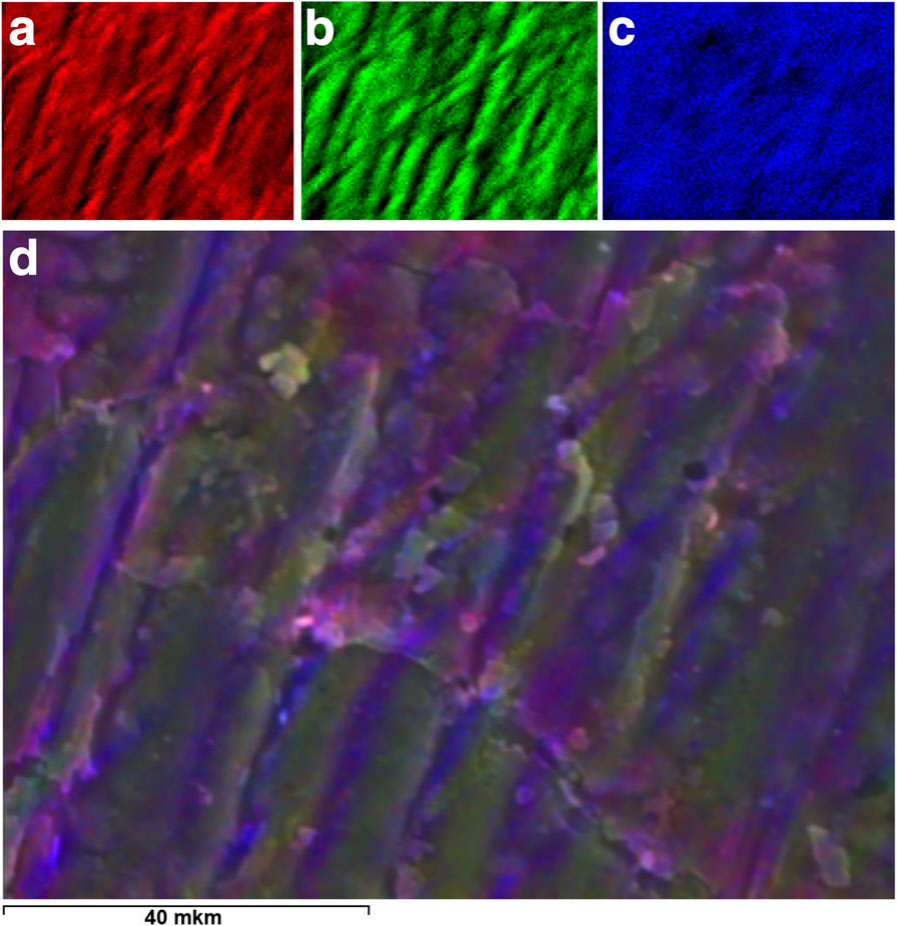
The elemental analysis results. The elemental map of the surface of the composite with 20 phr LiClO_4_: **a** C, **b** O, **c** Cl, and the **d** combined map

Summary elemental map (Fig. 9d) of the composite with 20 phr of LiClO_4_ was constructed from elemental maps of individual elements (carbon—Fig. 9a; oxygen—Fig. 9, b; chlorine—Fig. 9c) for determination of distribution of elements on its surface. The calculations have shown that the content of the elements on the surface of the composite are the following: carbon is 51.57 wt%, oxygen is 43.79 wt%, and chlorine is 4.64 wt%, while their distribution coincides with the ordering of inclusions identified by the means of optical and electron microscopy. This allows concluding that the nature of these inclusions with oxygen and chlorine saturation is inorganic.

## Conclusions

The comprehensive study of composites based on aliphatic epoxy oligomer (DEG-1) containing different amounts of LiClO_4_ salt (0–50 phr) revealed creation of interactions between lithium cations Li^+^ and the macromolecular chain of DEG-1 with forming of coordinative complexes. It is reflected in a substantial reduction of segmental mobility of DEG-1 chains within the formed complexes that linearly increase glass transition temperature *T*_g_ of polymer matrix with the salt content.

It was found that at higher temperature (200 versus 60 °C), the values of conductivity *σ* are three orders of magnitude higher with maximum at 15 phr of LiClO_4_. Such conductivity behavior is explained by existence of two opposite competitive processes, namely, the growth of salt content in composite gives the increase of carrier number and the raise of conductivity. On the other hand, the restriction of molecular movements of DEG-1 because of the forming of the coordinative complexes reduces the carrier mobility. At higher temperatures, the raising of molecular movements compensates this mechanism and conductivity becomes essentially higher.

Detailed IR spectroscopy study allowed suggesting the scheme of LiClO_4_ interaction with polymer chains, namely, the possible ion-dipole interactions of Li^+^ ion with the ether bond of polyethylene oxide fragment and OH group of the disclosed epoxy ring of DEG-1, with secondary amine group, or tertiary amine group of PEPA and ether bond simultaneously.

The results of morphological and structural studies by means of optical and electron microscopes as well as by elemental analysis have revealed the presence of inclusions with sizes from nanometers up to ~20 μm, probably, of inorganic nature distributed in the polymer matrix.

## Abbreviations

DEG-1: Epoxy oligomer of diglycide aliphatic ester of polyethylene glycol
DSC: Differential scanning calorimetry
IR: Infrared
LiClO_4_: Lithium perchlorate salt
PEO: Polyethylene oxide
PEPA: Polyethylene polyamine
ROM: Reflective optical microscopy
SEM: Scanning electron microscopy
SPE: Solid polymer electrolytes
*T*_g_: Glass transition temperature
TOM: Transmission optical microscopy
WAXS: Wide-angle X-ray spectroscopy
